# Body Weight and Body Mass Index Influence Bone Mineral Density in Late Adolescence in a Two‐Year Follow‐Up Study. The Tromsø Study: Fit Futures

**DOI:** 10.1002/jbm4.10195

**Published:** 2019-08-21

**Authors:** Ole Andreas Nilsen, Luai Awad Ahmed, Anne Winther, Tore Christoffersen, Gyrd Thrane, Elin Evensen, Anne‐Sofie Furberg, Guri Grimnes, Elaine Dennison, Nina Emaus

**Affiliations:** ^1^ Department of Health and Care Sciences The Arctic University of Norway Tromsø Norway; ^2^ Division of Neurosciences, Orthopedics and Rehabilitation Services University Hospital of North Norway Tromsø Norway; ^3^ Department of Health and Care Sciences Finnmark Hospital Trust, Alta Norway; ^4^ Department of Clinical Research University Hospital of North Norway, Tromsø, Norway, and Department of Health and Care Sciences, The Arctic University of Norway Tromsø Norway; ^5^ Department of Community Medicine The Arctic University of Norway Tromsø Norway; ^6^ Division of Internal Medicine University Hospital of North Norway, Tromsø, Norway, and Endocrine Research Group, Department of Clinical Medicine, The Arctic University of Norway Tromsø Norway; ^7^ MRC Lifecourse Epidemiology Unit, Southampton UK and Victoria University Wellington New Zealand; ^8^ Department of Microbiology and Infection Control Division of Internal Medicine University Hospital of North Norway Tromsø Norway

**Keywords:** PEAK BONE MASS, BMI, ADOLESCENCE, GENERAL POPULATION STUDIES, DXA

## Abstract

Determinants of bone acquisition in late adolescence and early adulthood are not well‐described. This 2‐year follow‐up study explored the associations of body weight (BW), body mass index (BMI), and changes in weight status with adolescent bone accretion in a sample of 651 adolescents (355 girls and 296 boys) between 15 and 19 years of age from The Tromsø Study: Fit Futures. This Norwegian population‐based cohort study was conducted from 2010 to 2011 and was repeated from 2012 to 2013. We measured femoral neck, total hip, and total body bone mineral content and areal bone mineral density (aBMD) by dual‐energy X‐ray absorptiometry. We measured height, BW, calculated BMI (kg/m ^2^), and collected information on lifestyle at both surveys. Mean BMI (SD) at baseline was 22.17 (3.76) and 22.18 (3.93) in girls and boys, respectively. Through multiple linear regression, baseline BW and BMI were positively associated with ∆aBMD over 2 years of follow‐up at all skeletal sites in boys ( *p* < 0.05), but not in girls. ∆BW and ∆BMI predicted ∆aBMD and ∆BMC in both sexes, but the strength of the associations was moderate. Individuals who lost weight during follow‐up demonstrated a slowed progression of aBMD accretion compared with those gaining weight, but loss of BW or reduction of BMI during 2 years was not associated with net loss of aBMD. In conclusion, our results confirm that adequate BW for height in late adolescence is important for bone health. Associations between change in weight status and bone accretion during follow‐up were moderate and unlikely to have any clinical implication on adolescents of normal weight. Underweight individuals, particularly boys, are at risk of not reaching optimal peak bone mass and could benefit from an increase in BMI. © 2019 The Authors. *JBMR Plus* is published by Wiley Periodicals, Inc. on behalf of the American Society for Bone and Mineral Research.

## Introduction

Osteoporosis is a major public health concern and a frequent cause of disability in Western societies.^1^ Norway has one of the highest reported hip fracture incidences in the world.^2^ Areal bone mineral density (aBMD) is a surrogate measure of bone strength and a strong predictor of fracture risk.^3^ Although genetics explain a substantial proportion of the variance of an individual's bone mass, lifestyle factors influence skeletal dynamics particularly during growth. Adolescence is a critical period for bone accretion and attainment of peak bone mass, defined as the highest bone mass obtained in a lifetime.^4^ Suboptimal acquisition of peak bone mass may lead to increased risk of osteoporosis and fragility fractures in later life.^5^, ^6^

It has long been established that there is an association between BW and bone mineral parameters in the adult population.^7^ High BMI is generally considered to have an osteo‐protective effect, while rapid loss of BW is associated with bone loss.^8^, ^9^ In childhood and adolescence, however, the relationship between weight status and bone accretion is more controversial. Both detrimental and protective effects of BW have been reported.^10^, ^11^, ^12^, ^13^, ^14^, ^15^, ^16^, ^17^, ^18^ There are few studies with repeated measures exploring bone accretion and longitudinal relationships.^16^ Obesity and overweight in childhood and adolescence are a growing concern worldwide with rising prevalence during the past decades.^19^ In European countries, including Norway, there has been a shift in the BMI distribution, with an increase in BMI in the upper percentiles.^20^ For health benefits, obese and overweight individuals are recommended to reduce their weight by approximately 10%. In older adults, evidence suggests that a weight reduction of that magnitude will induce a loss of bone of 1% to 2% and even up to 4% at highly trabecular sites such as the trochanter.^21^

Associations and interplay between anthropometric traits, aBMD levels, and bone accretion in late adolescence are not yet fully described and understood at a population level. The mechanisms behind the weight and bone relationship are not clear as both direct and indirect effects related to mechanical forces, nutrition, age, and hormonal status could be involved. The objectives of this 2‐year follow‐up population‐based study were to explore the associations between baseline BW, baseline BMI, changes in BW (∆BW), and changes in BMI (∆BMI) on changes in bone mineral parameters in a Norwegian population from 15 to 19 years of age. We hypothesized that higher baseline BW and BMI, as well as ∆BW and ∆BMI would be positively associated with changes in bone parameters, and that negative ∆BW and ∆BMI could be detrimental to bone accrual in adolescents entering young adulthood.

## Subjects and Methods

### Subjects

**Figure 1:**
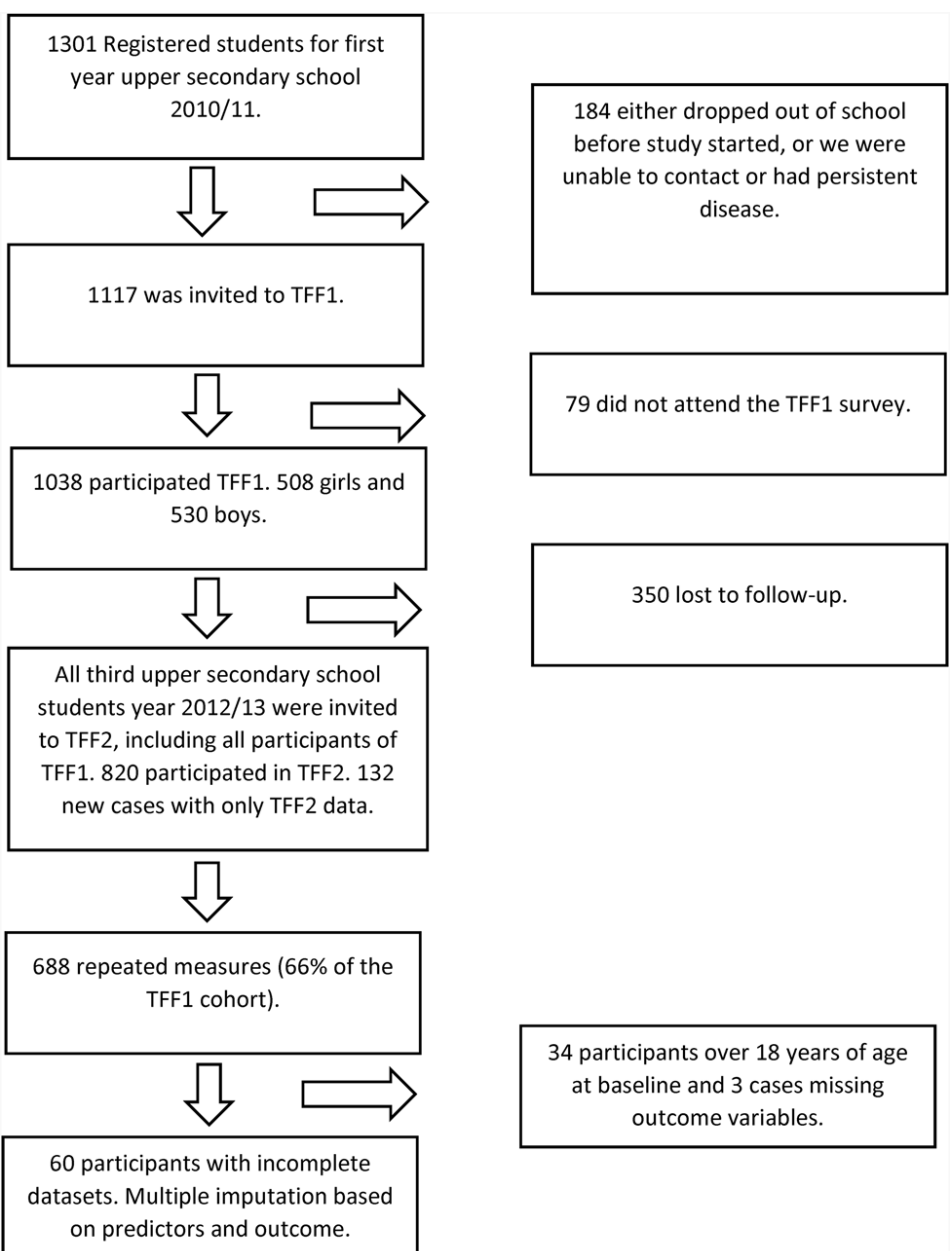
Flowchart of participation in Fit Futures 1 (TFF1) 2010 to 2011 and Fit Futures 2 (TFF2) 2012 to 2013. The Tromsø Study, Fit Futures.

Detailed information on the Fit Futures Study participants and study procedures has been published previously.^18^ Briefly, the Fit Futures study, an expansion of the Tromsø study in Northern Norway,^22^ invited all first year upper‐secondary school students (15 to 17 years of age) in Tromsø and the neighboring municipalities to a comprehensive health survey in 2010 to 2011 (TFF1). In this initial survey, 1117 participants were invited and 1038 adolescents (508 girls and 530 boys) attended (attendance rate of 93%). Two years later, in 2012 to 2013, we invited all TFF1 participants and all third‐year students in the same upper‐secondary schools to a follow‐up survey, Fit Futures 2 (TFF2), providing 688 repeated measures of aBMD (66% of the original cohort; Fig. 1). The Clinical Research Unit at the University Hospital of North Norway conducted both surveys during school days. The participants received information about the study in classrooms and all participants gave written informed consent at the study site. Participants younger than 16 years of age had to bring written consent from their guardians to take part in the survey. The data collection in TFF1 and TFF2 was approved by the Norwegian Data Protection Authority and the Regional Committee of Medical Research Ethics (REK nord) with project‐specific approval for the present study (Ref. 2013/1459/REK nord).

### Measurements

Femoral neck (FN), total hip (TH), and total body (TB) bone mineral content (BMC; g), bone area (BA; cm^2^) and aBMD (g/cm²) were measured by the same instrument (GE Lunar Prodigy; GE Lunar, Madison, WI, USA) by DXA and analyzed with enCORE pediatric software (GE Healthcare, Piscataway, NJ, USA)^23^ in both TFF1 and TFF2. We used auto‐analysis software and default region of interest, according to a standardized protocol. The primary outcome of the study was aBMD, but BMC and BA are reported to complement the understanding of bone accretion and growth. The precision of measurements expressed as coefficient of variation ([SD/mean] × 100) has previously been estimated to be 1.14% at the TH and 1.72% at the FN measured in vivo.^24^ We used measurements of the left hip. In 15 cases the left hip data were erroneous or missing and the right hip data were reported for both TFF1 and TFF2. We measured height and BW to the nearest 0.1 cm and 0.1 kg on a Jenix DS 102 Stadiometer (Dong Sahn Jenix, Seoul, Korea), following standardized procedures. BMI was calculated as BW divided by height squared (kg/m^2^), and participants were stratified into BMI quartiles. To explore if relationships changed with various BMI cut‐off points, we also categorized participants into underweight, normal weight, overweight, or obese. Participants <18 years of age were stratified based on their sex‐ and age‐specific BMI according to half‐year cut‐off points described by Cole and Lobstein.^25^ To describe the crude impact of change‐in‐weight status on bone accretion, we dichotomized participants into BMI losers and BMI gainers.

### Interviews and questionnaires

Information on ethnicity, the possibility of pregnancy (exclusion criterion for DXA), the presence of acute and chronic disease, and the use of medication and hormonal contraceptives was elicited by clinical interviews. We collected pubertal maturation information, perceived physical activity level, alcohol consumption, and tobacco use by electronic self‐administered questionnaires. Pubertal status for girls was determined based on age at menarche and answers were categorized into “Early” (<12.5 years at menarche), “Intermediate” (12.5 to 13.9 years), or “Late” (>14 years) pubertal maturation. We used the Pubertal Developmental Scale (PDS) to assess pubertal maturation in boys. Secondary pubertal characteristics such as growth spurt, pubic hair growth, changes in voice, and facial hair growth were rated on a scale from 1 (Have Not Begun) to 4 (Completed), were summarized, and then were divided by 4. We categorized a score <2 as “Have Not Begun”, 2 to 2.9 as “Barely Started”, 3 to 3.9 as “Underway,” and a score of 4 as “Completed.”^26^ Perceived physical activity level was assessed by a scale developed by Saltin and Grimby.^27^ The participants were asked to grade leisure time physical activity an average week during the last year with four alternatives: sedentary activities only; moderate activity like walking, cycling, or exercise at least 4 hours per week; participation in recreational sports at least 4 hours per week; and participation in hard training/sports competitions several times a week. Questions on smoking and snuffing had three alternatives: Never, Sometimes, or Daily. We assessed the frequency of alcohol consumption with a scale from 1 to 5: “Never,” “Once per Month or Less,” “2‐4 Times per Month,” “2‐3 Times per Week,” and “4 or More Times per Week.” Answers on the use of medication known to affect bone, presence of diseases known to affect bone, hormonal contraceptive use, smoking, snuff use, and alcohol consumption were dichotomized into “Yes” and “No.”

### Statistical analyses

All analyses were sex‐specific. Population characteristics are presented by BMI quartiles at baseline. Continuous variables are presented by means (SDs) and categorical variables by count (percentages). We compared BMI quartile groups by using one‐way ANOVA with Bonferroni correction and χ^2^ test. Welch's ANOVA with Games‐Howell post hoc procedure was used if equal variances assumption was violated. We computed annual bone‐ and anthropometric‐change variables to account for differences in time between baseline and follow‐up measures when describing change and in crude comparisons of groups. Student's *t* test was used to compare BMI losers and BMI gainers.

Associations between the exposure variables baseline BW, baseline BMI, ∆BW, and ∆BMI and outcomes FN, TH, and TB ∆aBMD and ∆BMC during follow‐up were assessed by multiple linear regression using the bone mineral follow‐up score as outcome and baseline score as a covariate (Y_2_ = β0 + β_1_Y_1_ + β_2_X_BW_ + β_3_…). Initially we conducted a crude univariate analysis. We then compared the results using change‐score analysis (Y_2_ – Y_1_ = β0 + β_1_X_BW_) and checking for consistency because baseline adjustments in change‐score analysis may introduce bias.^28^, ^29^ All adjusted models included baseline anthropometric measures, time between measurements, pubertal maturation, and perceived baseline physical activity level. Other variables previously known to be of clinical importance like ethnicity, alcohol consumption, smoking, snuff use, diagnosis known to affect bone, medication known to affect bone (see Table 1, and hormonal contraceptive use (all baseline measures) were then added as covariates using a backwards elimination strategy where *p* = 0.10 were used as cut‐off to enter or leave the model. Any covariate with *p* ≤ 0.10 in a final model was included in all final models. Based on this procedure, alcohol consumption and diagnosis known to affect bone were excluded. We fitted separate models for baseline‐ and change‐exposure variables. Models with ∆BW were adjusted for ∆height. We checked for confounding and plausible 2‐way interactions related to age, pubertal maturation, and baseline weight versus weight change relationships. Because of statistical significance (*p* < 0.05) we added interaction terms BW * menarche age and BMI * menarche age in corresponding baseline ∆aBMD FN models in girls. In boys, a significant interaction between ∆BMI * BMI was detected and included in three ∆BMI models: FN ∆aBMD, FN ∆BMC, and TB ∆BMC; ∆BW * BW was added to the ∆BW TB ∆BMC model. Interactions were further explored and visualized by graphs.

**Table 1:** Characteristics by BMI Quartiles at Baseline TFF1 (2010 to 2011). The Tromsø Study, Fit Futures

				BMI quartiles at baseline				
			Total	First quartile (*n* = 89)	Second quartile (*n* = 89)	Third quartile (*n* = 89)	Fourth quartile (*n* = 88)	*p* value
Girls (*n* = 355)	Age (years)		16.61 (0.387)	16.69 (0.44)	16.64 (0.36)	16.60 (0.38)	16.52 (0.35)	0.042
	Body height (cm)		165.03 (6.48)	165.77 (6.49)	165.92 (6.15)	164.65 (6.44)	163.95 (6.70)	0.127
	Body weight (kg)		60.37 (10.61)	51.31 (4.48)	56.59 (4.03)	60.87 (5.12)	72.97 (11.65)	<0.001
	BMI (kg/m^2^)		22.17 (3.76)	18.65 (0.76)	20.54 (0.48)	22.42 (0.62)	27.13 (3.97)	<0.001
	FN aBMD (g/cm^2^)		1.07 (0.12)	1.03 (0.11)	1.06 (0.13)	1.07 (0.13)	1.13 (0.11)	<0.001
	TH aBMD (g/cm^2^)		1.06 (0.13)	1.02 (0.11)	1.05 (0.13)	1.06 (0.13)	1.12 (0.11)	<0.001
	TB aBMD (g/cm^2^)		1.14 (0.08)	1.09 (0.06)	1.13 (0.07)	1.14 (0.07)	1.20 (0.06)	<0.001
	FN BMC (g)		4.91 (0.71)	4.62 (0.59)	4.82 (0.65)	4.89 (0.68)	5.31 (0.72)	<0.001
	TH BMC (g)		32.01 (4.84)	30.06 (4.31)	31.39 (4.48)	31.82 (4.51)	34.81 (4.84)	<0.001
	TB BMC (g)		2522.89 (387.38)	2256.31 (258.47)	2451.88 (266.57)	2528.10 (333.98)	2859.05 (407.61)	<0.001
	FN BA (cm^2^)		4.59 (0.34)	4.50 (0.35)	4.57 (0.29)	4.59 (0.33)	4.73 (0.37)	<0.001
	TH BA (cm^2^)		30.15 (2.33)	29.53 (2.26)	30.05 (1.83)	30.07 (2.40)	30.95 (2.58)	0.001
	TB BA (cm^2^)		2207.37 (233.59)	2061.63 (165.65)	2170.54 (157.77)	2211.85 (207.55)	2384.14 (262.91)	<0.001
	Ethnicity	White	347 (97.8%)	84 (94.4%)	89 (100%)	88 (98.9%)	86 (97.7%)	0.068
		Others	8 (2.2%)	5 (5.6%)	0 (0%)	1 (1.1%)	2 (2.3%)	
	Menarche age (*n* = 348)	Early	110 (31.0%)	17 (19.3%)	22 (24.7%)	35 (40.2%)	36 (41.4%)	0.002
		Intermediate	165 (46.5%)	42 (47.7%)	48 (53.9%)	39 (44.8%)	39 (44.8%)	
		Late	73 (20.5%)	29 (33.0%)	19 (21.3%)	13 (14.9%)	12 (13.8%)	
	Physical activity at baseline	Sedentary	42 (12.0%)	17 (19.1%)	9 (10.0%)	7 (7.9%)	10 (11.2%)	0.054
		Moderate	141 (39.5%)	36 (40.4%)	26 (28.9%)	35 (39.3%)	44 (49.4%)	
		Sports	110 (30.8%)	22 (24.7%)	36 (40.0%)	28 (31.5%)	24 (27.0%)	
		Competition	63 (17.6%)	14 (15.7%)	19 (21.1%)	19 (21.3%)	11 (12.4%)	
	Alcohol (yes)		262 (73.2%)	58 (65.2%)	68 (75.6%)	72 (80.0%)	64 (71.9%)	0.160
	Smoking (yes)		68 (19.0%)	13 (14.6%)	15 (16.7%)	22 (24.4%)	18 (20.2%)	0.349
	Snuffing (yes)		108 (30.2%)	22 (24.7%)	24 (26.7%)	33 (36.7%)	29 (32.6%)	0.282
	Hormonal contraceptives use (yes)		118 (33.0%)	24 (27.0%)	32 (36.0%)	32 (36.0%)	30 (25.4%)	0.532
	Medication known to affect bone (yes)^a^		8 (2.2%)	1 (1.1%)	3 (3.4%)	3 (3.4%)	1 (1.1%)	0.646
	Diagnosis known to affect bone (yes)^b^		4 (1.1%)	0	1 (1.1%)	3 (3.4%)	0	0.199

			Total	First quartile (*n* = 74)	Second quartile (*n* = 74)	Third quartile (*n* = 74)	Fourth quartile (*n* = 74)	*p* value
Boys (*n* = 296)	Age (years)		16.60 (0.37)	16.50 (0.38)	16.63 (0.38)	16.67 (0.33)	16.61 (0.36)	0.034
	Body height (cm)		177.25 (6.52)	177.30 (6.45)	177.12 (7.05)	177.56 (6.56)	177.00 (6.10)	0.957
	Body weight (kg)		69.81 (13.68)	57.10 (5.16)	64.43 (5.49)	71.46 (5.49)	86.26 (14.11)	<0.001
	BMI (kg/m^2^)		22.18 (3.93)	18.14 (.85)	20.50 (.60)	22.64 (.62)	27.45 (3.64)	<0.001
	FN aBMD (g/cm^2^)		1.11 (0.15)	1.01 (0.11)	1.12 (0.14)	1.13 (0.13)	1.19 (0.16)	<0.001
	TH aBMD (g/cm^2^)		1.12 (0.15)	1.02 (0.11)	1.12 (0.13)	1.15 (0.14)	1.20 (0.16)	<0.001
	TB aBMD (g/cm^2^)		1.18 (0.10)	1.10 (0.08)	1.18 (0.08)	1.20 (0.08)	1.24 (0.09)	<0.001
	FN BMC (g)		5.99 (0.99)	5.32 (0.75)	6.01 (0.87)	6.12 (0.90)	6.53 (1.04)	<0.001
	TH BMC (g)		40.17 (6.64)	35.61 (5.20)	40.11 (5.76)	41.30 (6.09)	43.65 (6.79)	<0.001
	TB BMC (g)		2963.78 (469.83)	2556.57 (340.84)	2877.81 (330.39)	3084.27 (385.23)	3336.46 (432.67)	<0.001
	FN BA (cm^2^)		5.38 (0.39)	5.29 (0.43)	5.37 (0.35)	5.40 (0.35)	5.48 (0.39)	0.024
	TH BA (cm^2^)		35.73 (2.47)	34.71 (2.61)	35.69 (2.31)	36.03 (2.21)	36.48 (2.47)	<0.001
	TB BA (cm^2^)		2496.46 (240.06)	2307.67 (189.40)	2443.49 (175.25)	2555.57 (201.59)	2679.11 (222.11)	<0.001
	Ethnicity	White	291 (98.3%)	74 (100%)	71 (95.9%)	74 (100%)	72 (97.3%)	
		Others	5 (1.7%)	0 (0%)	3 (4.1%)	0 (0%)	2 (2.7%)	
	Puberty development scale (*n* = 241)	Just started	22 (18.1%)	9 (16.7%)	12 (18.8%)	9 (14.3%)	14 (22.6%)	0.216
		Underway	177 (72.8%)	43 (79.6%)	49 (76.6%)	45 (71.4%)	40 (64.5%)	
		Completed	22 (9.1%)	2 (3.7%)	3 (4.7%)	9 (14.3%)	8 (12.9%)	
	Physical activity at baseline (*n* = 293)	Sedentary	77 (26.3%)	24 (32.9%)	10 (13.5%)	14 (19.2%)	29 (39.7%)	0.004
		Moderate	75 (25.6%)	21 (28.8%)	22 (29.7%)	16 (21.9%)	16 (21.9%)	
		Sports	70 (24.2%)	17 (23.3%)	20 (27.0%)	17 (23.3%)	17 (23.3%)	
		Competition	62 (23.9%)	11 (15.1%)	22 (29.7%)	26 (35.6%)	11 (15.1%)	
	Alcohol (yes)		195 (65.9%)	49 (66.2%)	41 (55.4%)	50 (67.6%)	55 (74.3%)	0.109
	Smoking (yes)		62 (20.9%)	19 (25.7%)	9 (12.2%)	16 (21.6%)	18 (24.3%)	0.173
	Snuffing (yes)		108 (36.5%)	30 (40.5%)	14 (18.9%)	30 (40.5%)	34 (45.9%)	0.003
	Medication known to affect bone (yes)^a^		6 (2.0%)	1 (1.4%)	2 (2.7%)	1 (1.4%)	2 (2.7%)	>0.999
	Diagnosis known to affect bone (yes)^b^		5 (1.7%)	1 (1.1%)	1 (1.1%)	2 (2.7%)	1 (1.1%)	>0.999

Continuous variables are described by mean (SD) and categorical by count (%).Cut‐off points for BMI quartiles (kg/cm2) were 19.71, 21.43, and 23.48 in girls and 19.39, 21.56, and 23.77 in boys.

^a^ Medication known to affect bone (ATC): D07A Plain corticosteroids, H03A Thyroid preparations, N03A Antiepileptic, R01AD Corticosteroids, R03BA Glucocorticoids (inhalants), and H02A Corticosteroids for systemic use.

^b^ Diagnosis known to affect bone (according to the 10th revision of the International Statistical Classification of Diseases and Related Health Problems): E03 Hypothyroidism, E10 Diabetes type 1, F50.9 Eating disorders, K90.0 Celiac disease, and M13 Arthritis.

aBMD = Areal bone mineral density; BMC = bone mineral content; BA = bone area; FN = femoral neck; TH = total hip; TB = total body; ATC = Anatomical Therapeutic Chemical.

Late introduction of the PDS questions in TFF1 may be the reason for a relatively high percentage of missing puberty values for boys: *n* = 53 (17.9%). Other missing covariates were menarche age in seven girls and physical activity in one girl and three boys. Multiple imputations based on predictors and outcome variables were performed to predict missing values. We assumed missing at random and 20 imputations were conducted,^30^ and we report pooled estimates. Normal distribution, linearity, homogeneity, and outliers were explored by residual analysis. In girls, two outliers were excluded in TH ∆aBMD: one in FN ∆aBMD and one in TH ∆BMC models. We used weighted least square regression in all TB ∆BMC models in girls to account for heteroscedasticity. Significance level was set to *p* = 0.05 and all procedures were performed in IBM SPSS Statistics for Windows, version 24 (IBM Corp., Armonk, NY, USA). Figures were made in RStudio (RStudio, Boston, MA, USA; (http://www.rstudio.com/)

## Results

### Descriptives

We included 651 adolescents with repeated measurements in the analyses, 355 girls and 296 boys (45.2% boys). At baseline, mean age was 16.6 years (range, 15.7 to 17.9), and 18.6 years (range, 17.8 to 20.1) at follow‐up. Average follow‐up time was 1.94 years (SD 0.2). Table 1 displays the baseline characteristics according to BMI quartile groups. In girls, mean group BMI for first to fourth quartile were 18.65, 20.54, 22.42, and 27.13 kg/m^2^, respectively. In boys, means were 18.14, 20.50, 22.64, and 27.45 kg/m^2^, respectively.

One‐way ANOVA analyses showed that cross‐sectional anthropometric, aBMD, and BMC measures differed significantly with a positive linear trend between BMI quartiles at baseline, except body height. These cross‐sectional differences persisted at follow‐up 2 years later (not shown). In girls, menarche age differed significantly with higher prevalence of early menarche at the two upper BMI quartiles compared with the bottom quartile (*p* = 0.002). In boys, physical activity at baseline differed significantly (*p* = 0.004) with a higher prevalence of sedentary behavior for the upper quartile (39.7%) compared with the other quartiles, and there was a low proportion of snuff users in the second quartile compared with the three other groups (*p* = 0.003).

Among girls, 5.9% of participants were classified as underweight, 75.4% normal weight, 14.0% overweight, and 4.7% obese according to Cole's weight classification at baseline with a mean group BMI of 17.6, 21.9, 26.2, and 33.9 kg/m^2^, respectively. Among boys, 8.4% of participants was classified as underweight, 70.6% normal weight, 14.5% overweight, and 6.4% as obese. Mean group BMI in boys were 17.2, 21.0, 26.2, and 32.5 kg/m^2^, respectively. Proportions in the two upper categories increased during follow‐up. In girls, the prevalence of overweight and obesity combined had increased to 20.6% in 2 years. In boys, the prevalence of overweight and obesity combined increased to 28% at TFF2 (data not shown).

In girls, mean annual BW and BMI change was 1.38 kg (95% confidence interval [CI], 1.12 to 1.64) and 0.41 kg/m^2^ (95% CI, 0.31 to 0.50). Boys gained 2.70 kg (95% CI, 2.35 to 3.04) and 0.61 kg/m^2^ (95% CI, 0.51 to 0.72), respectively. Eighty‐eight girls (24.6%) and 48 boys (16.2%) lost BW with an average annual loss of –1.60 (95% CI, –1.92 to –1.28) and –1.97 (95% CI, –2.43 to –1.51) kg. One‐hundred eleven girls (31.3%) and 62 boys (20.9%) reduced their BMI during follow‐up, with a mean annual decrease of −0.56 (95% CI, –0.66 to –0.46) and –0.66 (95% CI, –0.81 to –0.51) kg/m^2^. We observed a clear difference in longitudinal growth between girls and boys. In girls, 280 (78.9%) of the participants had an increment in height between measurements with an annual mean of 0.053 cm (95% CI, 0.049 to 0.056). Almost all the boys (93.2%, *n* = 276) grew taller during the 2 years of follow‐up. Annual mean change was 1.024 cm (95% CI, 0.928 to 1.120).

**Figure 2:**
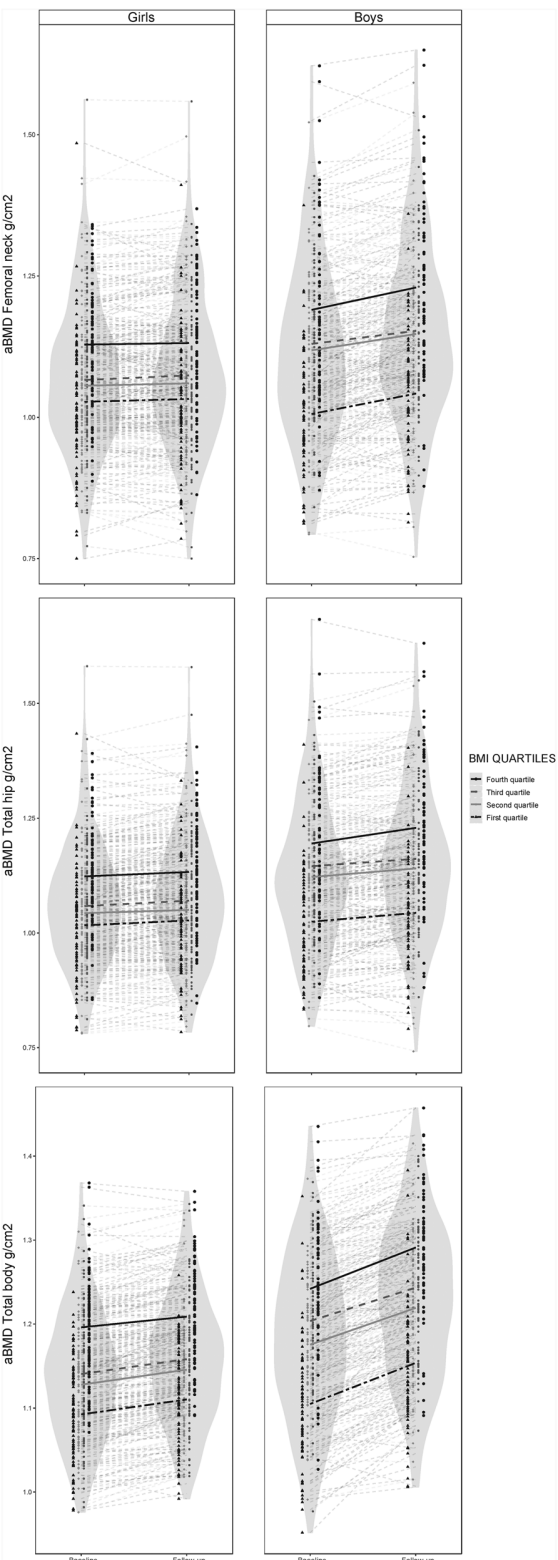
Femoral neck‐, total hip‐, and total‐body aBMD in girls and boys from TFF1 (2010 to 2011) to TFF2 (2012 to 2013). Individual measures and group mean according to BMI quartiles at baseline. Girls, *n* = 355. Boys, *n* = 296. The Tromsø Study, Fit Futures. In girls, cut‐off points for BMI quartiles were 19.7, 21.4, and 23.5 and in boys 19.4, 21.6, and 23.8, respectively. The grey area (violin plot) shows the full population distribution at TFF1 and TFF2 in both girls and boys. The points specify each individual measurement and the thin dotted lines show participants individual accretion during follow‐up. The thick lines indicate the baseline BMI quartile group mean aBMD accretion between measurements. aBMD = Areal bone mineral density; BMI = body mass index (kg/m^2^).

Cross‐sectional measures and the individual aBMD trajectories from TFF1 to TFF2 and unadjusted means within baseline BMI quartiles are illustrated in Fig. 2. Post hoc analysis showed that, among boys, the first quartile had significantly lower, and the fourth quartile significantly higher FN, TH, and TB aBMD than the other quartiles at both time points (*p* < 0.05). There were no significant differences in aBMD status between second and third quartiles in any of the three skeletal sites, neither at baseline nor at follow‐up. In girls, the pattern appeared similar to boys, but less polarized in the lower BMI quartiles. The aBMD levels in girls in the first quartile did not differ significantly from the two middle quartiles at the femoral sites.

**Figure 3:**
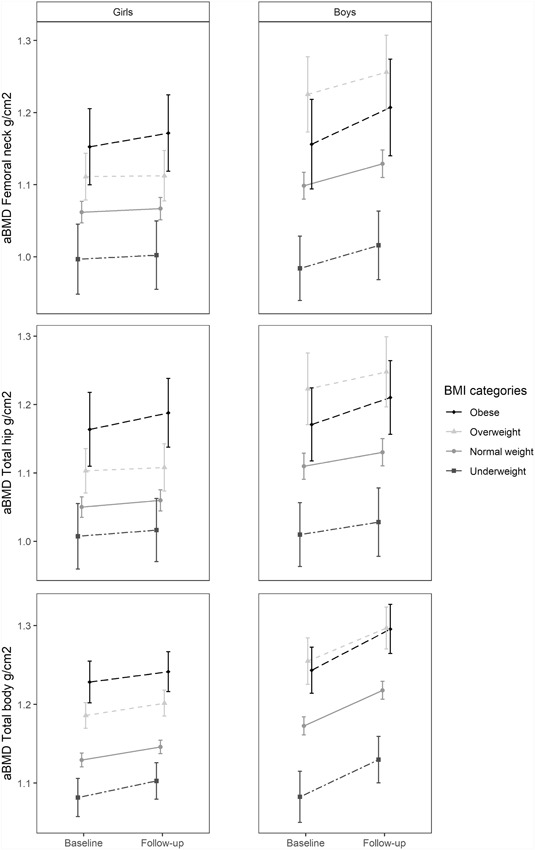
Mean aBMD accretion of femoral neck, total hip and total body aBMD in girls and boys between baseline survey TFF1 (2010 to 2011) and the follow‐up survey TFF2 (2012 to 2013) according to baseline BMI categories underweight, normal weight, overweight, and obese. The Tromsø Study, Fit Futures. Girls, *n* = 355. Boys, *n* = 296. In girls, the BMI intervals for baseline BMI categories were: underweight: 16.2 to 18.2, normal weight: 18.1 to 24.7, overweight: 24.5 to 29.1, and obese: 29.8 to 41.2 (kg/cm^2^). In boys, the intervals were 16.2 to 17.8, 17.7 to 24.2, 24.2 to 28.9, and 29.6 to 40.3 (kg/cm^2^), respectively. Error bars = 95% confidence interval. aBMD = Areal bone mineral density; BMI = body mass index (kg/cm^2^).

When participants were stratified into BMI categories, the relationships slightly changed. Figure 3 indicates that although not statistically significant, and unlike the girls, boys in the obese category had lower mean FN, TH, and TB aBMD at both measure points compared with their overweight peers. Boys classified as underweight had significantly lower aBMD at baseline compared with those with normal weight (FN: *p* = 0.001, TH: *p* = 0.005, TB: *p* < 0.001) and this pattern persisted during the 2 years of follow‐up in crude analyses.

### Body weight, body mass index, and bone accretion

Changes in anthropometry, ∆aBMD, and ∆BMC during follow‐up according to baseline BMI quartiles are presented in Table 2. In crude comparisons of quartiles, no statistically significant differences were found, except ∆BW, ∆BMI, and ∆TB BMC in girls. The first quartile gained more weight compared with the second quartile, and accumulated more total body bone than the fourth quartile.

**Table 2:** Annual change in body height (cm), body weight (kg), BMI (kg/m^2^), aBMD (g/cm^2^) and BMC (g) between TFF1 (2010‐2011) and TFF2 (2012‐2013) by BMI quartiles at baseline. The Tromsø Study, Fit Futures

			*BMI quartiles at baseline*				
		Total	First quartile	Second quartile	Third quartile	Fourth quartile	p‐value
Girls (n = 355)	∆ Body height	0.365 (0.455)	0.419 (0.521)	0.389 (0.388)	0.325 (0.435)	0.326 (0.455)	0.417
	∆ Body weight	1.383 (2.501)	1.928 (1.718)^2^	0.934 (1.944)^1^	1.275 (2.238)	1.394 (3.614)	0.004
	∆ BMI	0.406 (0.910)	0.608 (0.656)^2^	0.243 (0.699)^1^	0.381 (0.852)	0.392 (1.280)	0.005
	∆ FN aBMD	0.003 (0.019)	0.003 (0.018)	0.003 (0.019)	0.005 (0.020)	0.002 (0.018)	0.755
	∆ TH aBMD	0.005 (0.017)	0.006 (0.017)	0.004 (0.018)	0.006 (0.017)	0.005 (0.016)	0.809
	∆ TB aBMD	0.009 (0.010)	0.009 (0.010)	0.010 (0.009)	0.009 (0.011)	0.006 (0.010)	0.094
	∆ FN BMC	0.014 (0.095)	0.011 (0.093)	0.017 (0.093)	0.017 (0.096)	0.013 (0.099)	0.970
	∆ TH BMC	0.180 (0.592)	0.241 (0.605)	0.116 (0.557)	0.171 (0.626)	0.193 (0.580)	0.563
	∆ TB BMC	39.609 (60.362)	55.290 (37.087)^4^	32.229 (50.509)	45.379 (52.165)	25.379 (86.922)^1^	0.001
Boys (n = 296)	∆ Body height	0.929 (0.867)	1.076 (1.011)	0.896 (0.619)	0.864 (1.103)	0.882 (0.624)	0.414
	∆ Body weight	2.697 (3.022)	2.928 (2.332)	2.974 (2.413)	2.661 (3.370)	2.224 (3.732)	0.481
	∆ BMI	0.614 (0.950)	0.692 (0.718)	0.713 (0.736)	0.629 (1.082)	0.424 (1.170)	0.315
	∆ FN aBMD	0.16 (0.027)	0.018 (0.025)	0.015 (0.026)	0.013 (0.028)	0.020 (0.028)	0.402
	∆ TH aBMD	0.012 (0.022)	0.010 (0.022)	0.010 (0.023)	0.009 (0.023)	0.017 (0.022)	0.093
	∆ TB aBMD	0.023 (0.015)	0.024 (0.016)	0.022 (0.015)	0.021 (0.015)	0.024 (0.016)	0.475
	∆ FN BMC	0.100 (0.176)	0.107 (0.173)	0.089 (0.175)	0.077 (0.176)	0.129 (0.180)	0.308
	∆ TH BMC	0.566 (1.072)	0.514 (1.067)	0.527 (1.177)	0.440 (1.029)	0.783 (0.997)	0.229
	∆ TB BMC	118.818 (77.247)	121.371 (67.240)	121.005 (69.577)	118. 124 (81.233)	114.773 (90.133)	0.951

aBMD =Areal bone mineral density (g/cm^2^), BMC = Bone mineral content (g), FN = Femoral neck, TH = Total hip, TB = Total body, BMI = Body mass index (kg/cm^2^), body weight in kg, ∆  = change. Cut‐offs points for BMI quartiles were 19.71, 21.43, 23.48 (kg/m^2^) in girls and 19.39, 21.56, 23.77 (kg/m^2^) in boys. Average follow‐up time was 1.94 years (SD 0.2).^1234^ Significantly different from specified quartile (p  <  0.05) analysed using bonferroni post‐hoc test for multiple comparisons.

**Figure 4:**
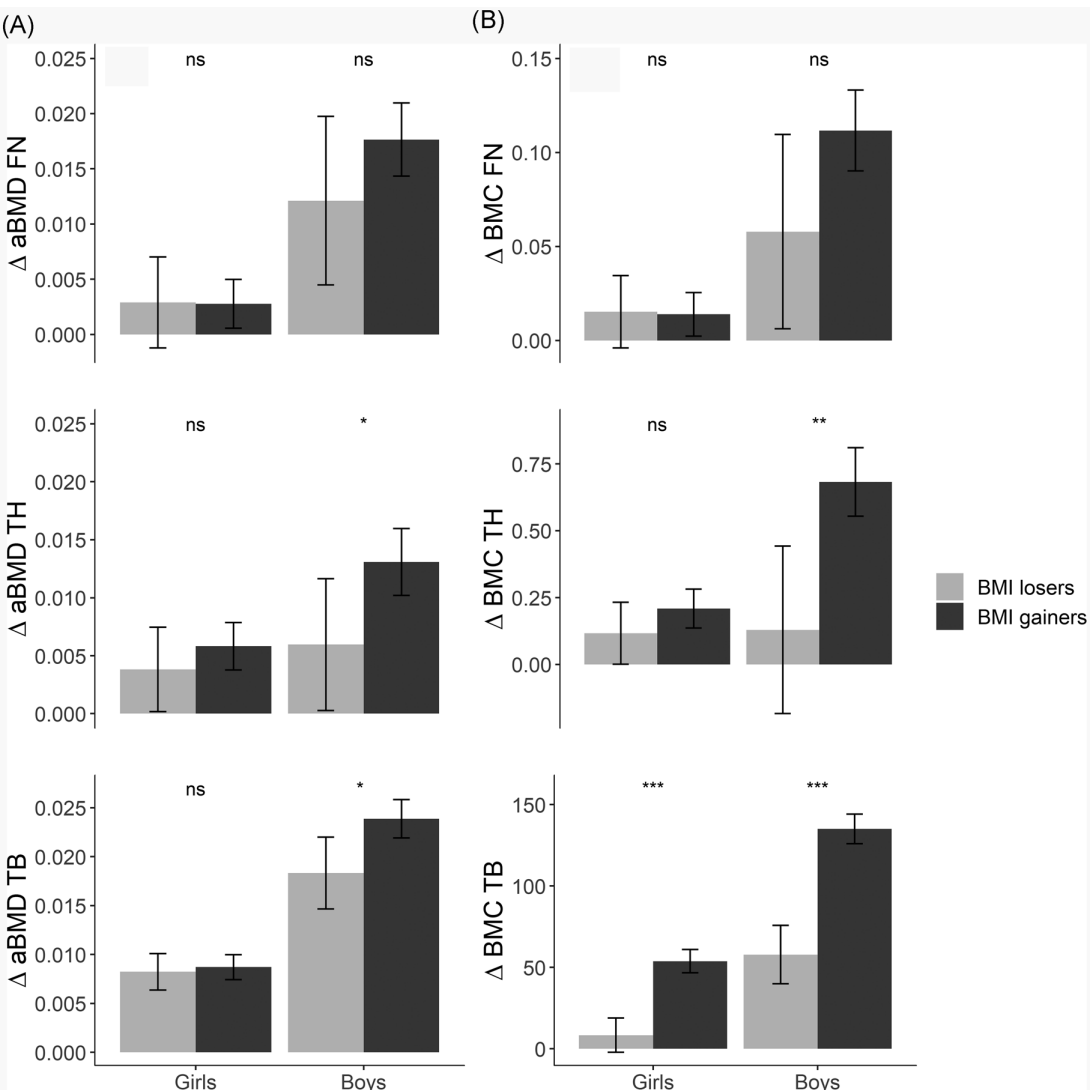
Mean annual (*A*) aBMD and (*B*) BMC change in BMI losers and BMI gainers between baseline survey TFF1 (2010 to 2011) and the follow‐up survey TFF2 (2012 to 2013). The Tromsø Study, Fit Futures. Girls, *n* = 355. Boys, *n* = 296. BMI loser girls: *n* =111, BMI losers boys: *n* = 62. FN = Femoral neck; TH = total hip; TB = total body; ∆aBMD = change in areal bone mineral density (g/cm^2^); ∆BMC = change in bone mineral content (g); BMI = body mass index (kg/cm^2^). Error bars = 95% confidence interval. Two‐tailed *t*‐test for differences in mean: ns: *p* > 0.05, *p ≤ 0. 05, **p ≤ 0.01, ***p ≤ 0.001.

Figure 4 depicts mean ∆aBMD (Fig. 4 *A*) and ∆BMC (Fig. 4 *B*) among BMI losers and BMI gainers between TFF1 and TFF2. Reduction of BMI seemed to induce a slower bone accretion rate, especially in boys, but no mean bone loss was observed in any BMI loser group in either girls or boys. Among girls, statistically significant differences between the two groups were found only at TB ∆BMC (*p* < 0.001). Among boys, TH ∆aBMD (*p* = 0.027), TB ∆aBMD (*p* = 0.011), FN ∆BMC (*p* = 0.033), TH ∆BMC (*p* < 0.001), and TB ∆BMC (*p* < 0.001) were significant. The same pattern was observed with loss of BW. In boys, the BW loser group (*n* = 48) had a mean annual increment in TH aBMD of 0.006 g/cm^2^ (95% CI, 0.000 to 0.012); the BW gainers had a mean of 0.012 g/cm^2^ (95% CI, 0.010 to 0.015; not shown).

**Figure 5:**
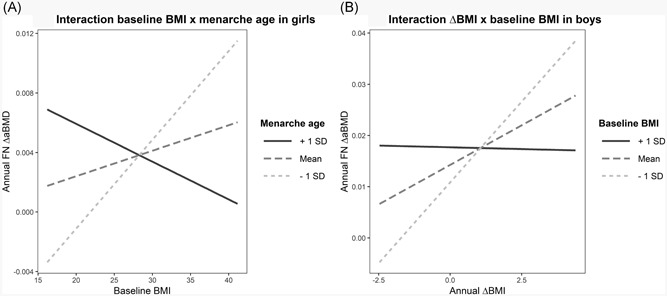
Visualization of interactions (*A*) baseline BMI and menarche age in girls and (*B*) baseline BMI and ∆BMI in boys in femoral neck ∆aBMD regression models. The Tromsø Study, Fit Futures. Girls: *n* = 354. Boys: *n* = 296. Interaction plots show unadjusted relationships from linear regression models, but the interactions persisted after adjustments of relevant confounders. Menarche age: mean (SD) =12.98 (1.19), baseline BMI in boys: mean (SD) = 22.18 (3.93). aBMD = Areal bone mineral density (g/cm^2^); BMI= body mass index (kg/m^2^); ∆ = change.

The crude and adjusted associations from multiple linear regression models between baseline BW, baseline BMI, ∆BW, ∆BMI, and ∆aBMD and ∆BMC are presented in Table 3. In girls, no associations between baseline measures and ∆aBMD were identified, but both baseline BW (*p* = 0.009) and baseline BMI (*p* = 0.021) were significantly associated with ∆BMC in the adjusted TH models. In boys, baseline BW and BMI were statistically significant predictors of both ∆aBMD and ∆BMC in most models. Exceptions were crude FN ∆aBMD/∆BMC and TB ∆BMC. ∆BW and ∆BMI had a consistent positive association with both ∆aBMD and ∆BMC in all adjusted models, except ∆BMI ∆aBMD TH (*p* = 0.086). The influence on ∆aBMD was strongest at femoral sites in boys, but overall changes in aBMD were moderate considering the size of the units of exposure. A baseline BMI difference of 1 SD (3.93 kg/m^2^) was associated with a 0.008 g/cm^2^ difference in TH ∆aBMD over 2 years (*p* = 0.002), whereas 1 SD ∆BMI (1.89 kg/m^2^) during follow‐up was associated with 0.004 g/cm^2^ ∆aBMD (*p* = 0.086). Statistically significant interactions were detected in six models. Pubertal maturation moderated the relationship of baseline BW/BMI and FN ∆aBMD in girls, whereas initial BW and BMI appeared to influence some of the change in weight–bone accretion associations in FN and TB among boys. The relationships between bone accretion and weight change were strongest among boys with low BMI/BW at baseline (Table 3, Fig. 5 *A* and Fig. 5 *B*).

**Table 3:** Adjusted associations between baseline and changes in weight parameters and femoral bone development during two year follow‐up. The Tromsø Study, Fit Futures

			FN				TH				TB			
			Crude		Adjusted		Crude		Adjusted		Crude		Adjusted^¤^	
			β	p	β	p	β	p	β	p	β	p	β	p
Girls n = 355	∆aBMD	Body weight	.003	.099	.001^*^	.669	.003	.116	.003	.189	.002	.184	.000	.971
		Body weight x menarche age			−.003	.013								
		BMI	.001	.546	.001^*^	.779	.002	.335	.002	.200	.000	.925	−.001	.607
		BMI x menarche age			−.003	.009								
		∆ Body weight	.004	.057	.002	.002	.005	.005	.005	.004	.002	.026	.002	.083
		∆ BMI	.001	.560	.001	.001	.004	.030	.004	.016	.002	.110	.001	.169
	∆BMC	Body weight	.024	.029	.019	.105	.171	.013	.182	.009	9.891	.294	7.074	.461
		BMI	.009	.378	.010	.339	.112	.076	.148	.021	−3.405	.642	−1.900	.803
		∆ Body weight	.026	.008	.024	.009	.221	<.001	.218	<.000	64.494	<.001	66.417	<.000
		∆ BMI	.015	.125	.021	.025	.181	.002	.287	.001	60.323	<.001	63.387	<.000

			FN				TH				TB			
			Crude		Adjusted		Crude		Adjusted		Crude		Adjusted	
			β	p	β	p	β	p	β	p	β	p	β	p
Boys n = 296	∆aBMD	Body weight	.008	.005	.009	.009	.008	.005	.009	.002	.006	.002	.006	.005
		BMI	.006	.076	.008	.008	.006	.021	.008	.002	.004	.024	.005	.007
		∆ Body weight	.007	.015	.004	.004	.008	.002	.005	.023	.008	<.001	.007	.003
		∆ BMI	.003	.333	.005^#^	.081	.004	.083	.004	.086	.006	.001	.006	.001
		∆ BMI x BMI			−.008	.004								
	∆BMC	Body weight	.059	.009	.078	.001	.072	.023	.374	.005	33.515	.008	34.190	.007
		BMI	.051	.017	.070	.001	.268	.043	.399	<.000	16.358	.130	18.815	.085
		∆ Body weight	.064	.001	.040	.030	.548	<.001	.328	.005	93.669	<.001	87.503^§^	.000
		∆ Body weight x body weight											−19.109	.004
		∆ BMI	.031	.120	.049^#^	.017	.347	.005	.452	.002	77.863	<.001	84.189^#^	.000
		∆ BMI x BMI			−.056	.003							−24.348	.001

All β coefficients are per SD change in exposure. BMC = Bone mineral content (g), FN = Femoral neck, TH = Total hip, TB= Total body, BMI= Body mass index (kg/m^2^), body weight in kg. ∆ = change. adjusted models included age, sexual maturation, physical activity level, baseline aBMD or BMC measurement, time between measurements, ethnicity, use of medication known to affect bone, hormonal contraceptives use (girls), snuff use and smoking. In girls, one outlier in FN ∆aBMD (n = 354) models was excluded, two in TH ∆aBMD (n = 353) and one in TH ∆BMC models (n = 354). All baseline body weight models were adjusted for baseline height. ∆Body weight models were adjusted for baseline height and ∆ height, whereas ∆ BMI models adjusted for baseline BMI. Multiple imputation were conducted based on predictors and outcome variables in the adjusted models and pooled estimates are shown. ¤ Weighted least square regression (n = 348 because imputation were not used). *The effect of weight and BMI should be measured as (β1 + β3 (menarche age)), ^#^The effect of ∆ BMI should be measured as (β1 + β3 (BMI)), § The effect of ∆ body weight should be measured as (β1 + β3 (body weight)). All interactions are based on mean‐centered variables and visually explored in Figure 5.

## Discussion

In this population‐based study we explored the associations between BW, BMI, ∆BW, and ∆BMI with changes in bone parameters in adolescents entering young adulthood. Underweight boys had significantly lower mean aBMD at baseline and this disadvantage persisted during 2 years of follow‐up. Change in BW and BMI appeared to be a significant predictor of aBMD change for both girls and boys in the adjusted models, but the increments of aBMD for each unit change in exposure were relatively modest. Findings suggest that the influence of weight change might be strongest among boys with low BMI. Loss of BW or reduction of BMI was not associated with net loss of aBMD; however, our results indicate that the bone accretion rate slowed down whenever weight was lost or BMI reduced during follow‐up in both sexes. In the present study, more than one of five adolescents was classified as overweight or obese at baseline; the prevalence increased during follow‐up for both girls and boys.

The results supported our initial hypothesis with a few exceptions. In girls, the influence of baseline weight status on ∆aBMD was limited compared with the results in boys. This may be caused by gender differences in maturation. Cessation of longitudinal growth in girls and strong genetic control reduce the accumulation of bone mass. Previously published results indicate that girls reach a femoral aBMD plateau between 17 and 19 years of age.^18^ The influence of baseline BW and BMI may therefore be less in girls in this age interval because adaptation to mechanical loading is greater in a growing skeleton.^31^

A positive cross‐sectional association between BMI and aBMD and a positive association between baseline BW and increased *Z*‐score in femoral sites over 2 years in boys have previously been shown in the Fit Futures cohort.^18^, ^32^ In the present study, we report that cross‐sectional associations between BMI and aBMD were still present at TFF2 in both girls and boys 2 years later. Our findings are in accordance with a recent meta‐analysis and systematic review by Van Leeuwen and colleagues.^16^ They included 27 observational studies on the relationship between BW and bone mineral parameters in participants between 2 to 18 years of age and concluded that overweight and obese individuals had significantly higher aBMD and BMC than counterparts with normal BW. However, only one longitudinal study exploring the long‐term consequences of childhood obesity was included in the meta‐analysis. Threshold effects of BMI´s positive influence on bone have been previously reported.^33^, ^34^ Although nonsignificant and based on a small number of subjects, we observed that adolescent boys classified as overweight had the highest mean aBMD, higher than those classified as obese. This pattern was not observed when participants were stratified into BMI quartiles. The mean BMI was higher in the obese category (32.5 kg/m^2^) than the fourth BMI quartile (27.1 kg/m^2^), representing the tail of the distribution. In girls, the associations between baseline BMI categories and measured bone traits were positive and had a linear trend.

### Change in body weight and BMI and accretion of aBMD

Bone loss during weight reduction is well‐documented in older individuals, but not yet demonstrated in younger populations.^21^ We found no net loss of aBMD or BMC in participants losing BW or reducing their BMI during follow‐up. However, mean annual BMI reduction was modest (−0.56 kg/cm^2^ among girls and −0.66 kg/cm^2^ among boys) over 2 years in our study. To investigate more extreme cases of weight loss, an elaborate analysis stratifying ∆BMI in deciles was conducted (within 10th percentile, mean annual ∆BMI of −1.16 kg/m^2^ in both girls and boys), but a significant loss of aBMD was still not detected (not shown). The association between weight loss and loss of bone is more consistent in older compared with younger individuals.^35^ This may be linked to relatively better maintained muscle function in the younger age groups.^21^ There is a strong relationship between lean mass and bone, and healthy adolescents are less vulnerable to loss of muscle function during weight reduction compared with older peers. Furthermore, older people may be more prone to bone loss because of reduced efficiency in calcium absorption with age.^36^

The determinants of bone acquisition in the period of late adolescence to early adulthood are understudied,^4^ and there are a limited number of studies of weight change and bone in a comparable population. Most studies are among pre‐, peri‐, and postmenopausal women, in relation to weight‐reduction interventions, eating disorders, use of medications, or bariatric surgery.^21^, ^37^, ^38^ Studies on anorexia nervosa in adolescence are not directly comparable, but longitudinal studies of weight gain and restoration of BW show significant, although slow, improvement and normalization of aBMD levels.^39^ In a recent study, extensive BMI gain during puberty was associated with lower increments in aBMD.^40^ Exploring the effect of weight change on bone mass in obese female adolescents, Rourke and colleagues^41^ found no bone loss, but concluded that reduction of BW induced a reduced bone growth rate over 12‐month follow‐up—results that are comparable to our findings.

The effect of weight reduction on bone depends on whether it is voluntary or involuntary, the rate of change, age, sex, and initial weight.^37^ In the current study, we had no information on the reason for our participants’ BW reduction, whether it was based on dieting, disease/illness, or natural fluctuations. Normally, adults’ BW fluctuates by >0.25 kg/year, but in adolescence BW may be more unstable.^42^ Furthermore, we have no information on when during the 2‐year follow‐up the weight change occurred. The adaptive response delay of bones makes interpretations harder. Changes in weight precede skeletal adaptation to mechanical loads; the bone mass adaptation rate seems to depend on direction and magnitude as changes are more rapid during unloading than reloading.^8^ Bone adaptation to weight change has also been shown to be modified by exercise, nutrition, and medication.^21^ Compared with high initial body weight, leaner individuals have been demonstrated to suffer greater bone loss during weight reduction.^21^ We detected a statistically significant interaction between baseline BMI and ∆BMI in ∆aBMD FN model in boys indicating that the relationship between ∆BMI and bone accretion were strongest in boys with low BMI at baseline. In a crude analysis, this could very well be participants in the first quartile “catching up” based on age and pubertal maturation, but the relationship persisted after adjustments and the interaction was still present in the fully adjusted model. This interaction is potentially interesting; however, associations and relationships need to be tested and confirmed in other cohorts.

BMI reflects both muscle and adiposity; the mechanisms behind the relationship between weight status and bone are complex and multifactorial. Excess weight may have both negative and positive influences on bone health through different mechanisms. The process of bone modeling is sensitive to mechanical loading: It has been stated that high BW improves bone mineralization by increasing the forces applied on weight‐bearing bones.^43^ This effect has similarities to the positive effect of weight‐bearing physical activities on bones.^16^, ^44^ Both weight‐bearing activity and excess BW could lead to more lean mass. Greater lean mass, in addition to compressive force, produces increased tensile force on bone load and muscles produce the largest physiologic force on bone.^45^ Results in our study indicate that, in girls, weight‐based (and weight‐bearing‐based) interventions to maximize the genetic potential of peak bone mass at femoral sites should be implemented before the age of 15 years to be most effective. This is in agreement with studies indicating that prepuberty is the best time to change bone mass trajectory.^46^

On the other hand, weight‐bearing activity is essential during growth and excess BW may be associated with sedentary behavior (in the present study, 39.7% of the boys reported to be sedentary in the upper BMI quartile). In addition to the mechanical‐loading factors, adipose tissue may exert an impact on bone homeostasis and bone turnover through various adipokines like leptin and estrogen.^15^ Mechanisms behind the correlation between changes in weight and bone changes in older populations are proposed to be related to estrogen bioavailability or/and decreased calcium intake. Studies showing a reduction of BMC in the distal forearm during dietary weight reduction suggest hormonal aspects are involved, not just gravity and a response to weight‐bearing related forces.^47^ There is also evidence suggesting that obesity may influence the timing of puberty. Dimitri and colleagues^10^ highlight the effect of sex‐related changes in body composition when studying relationships between bone and body size. Obese children reach peak height velocity earlier than age‐matched lean children do, and late menarche is a determinant of lower aBMD and a known risk factor for fractures later in life.^48^ Thus, an early menarche in obese girls may have a long term osteoprotective effect. In the present study, menarche age moderated the baseline BMI versus FN ∆aBMD relationship. Among girls with self‐reported late menarche age, BMI appeared to be negatively correlated with FN ∆aBMD during follow‐up. This interaction was, however, partly driven by a few individuals with baseline BMI >35 with considerable regression line leverage, and the statistical significance of interaction attenuated (*p* = 0.083) when these participants were excluded in a sensitivity analysis.

### Strengths and limitations

The population‐based design and repeated measures from a well‐described representative sample of both sexes from different municipalities gave strengths to the present study. The sample size provided an opportunity to analyze the results in smaller subsamples, and explorations of the tails of the distribution are of clinical interest. Using a dedicated research unit at the University Hospital of North Norway ensured the high quality of the data acquisition. We used the same densitometer through both surveys, with continuous validations following a standardized common protocol. The main limitations of this study were the short follow‐up period of 2 years and that individuals were only measured twice. Short follow‐up periods increase the risk of being obscured by variability in DXA measurements. On the other hand, the recommended minimum interval between DXA scans is 6 to 12 months.^49^ Difference scores with two time points have limitations when exploring growth and development processes because the shape of the trajectory is unknown and additional measures would be preferred.^50^ There are different approaches when assessing correlates of change between two time points. Difference‐score as outcome (Y_2_ – Y_1_) and follow‐up measurement (Y_2_) as outcome using baseline (Y_1_) as a covariate are two frequently used methods. Authors recommend a comparison of methods for agreement because in some situations these two approaches can lead to a different conclusion in nonrandomized studies based on the statistical phenomenon regression to the mean and Lord's paradox.^29^, ^51^ We found agreement in femoral ∆aBMD models, but discrepancy in some of the TB and BMC associations (Supplemental Table S2). Thus, results from the multiple regression model concerning some of the TB and BMC in this study should be interpreted with caution. Nevertheless, discrepancies may also be explained by the fact that dissimilarities in models as difference‐scores without baseline adjustment fail to take the initial aBMD or BMC levels into account, consequently addressing slightly different concepts.

The 2D areal DXA measures have a tendency of overestimating BMC of larger bone because wider bones are also thicker; hence, the interpretation of measures of growing skeletons must be done with caution because of this size dependency.^52^ This concern especially applies to our male participants still experiencing longitudinal growth. Shape, body habitus, and changes in body composition may affect DXA measurements; it has been suggested that DXA may not be a valid technique for evaluating bone/weight associations.^53^ The impact of thickness of body tissue overlaying the measured area could be a concern in longitudinal studies of the effect of BW changes.^54^, ^55^ However, this mainly applies to lateral scans not performed in this study^56^, ^57^ and weight loss <6 kg has been shown to have limited influence on DXA aBMD measures.^37^ Dietary intake information such as calcium intake and vitamin D levels may play a role in bone accretion. Unfortunately, information on nutrition was not available in The Fit Future study. Changes during follow‐up in some of the control variables, such as increased proportions of smokers and snuff users, make the interpretations of associations harder (Supplemental Table S1). Nonparticipation and loss to follow‐up bias could be a problem. With the high attendance rate of 93% of those invited at baseline, the nonparticipation exposition is limited. Drop‐out analysis showed a higher proportion of boys, smokers, snuff users, and consumers of alcohol (girls) among the 32% lost at follow‐up compared with those who participated in both surveys. Girls lost at follow‐up had a moderately higher mean baseline BMI (*p* = 0.053). This could lead to underestimation of the association between BMI and bone accretion found in this study.

In conclusion, our results indicate that weight status during late adolescence could play a part in the concept of maximizing bone mass and density during growth for prevention of future fractures. ∆BW and ∆BMI predicted ∆aBMD and ∆BMC in both sexes. Although statistically significant, the magnitude of these changes in aBMD during follow‐up was moderate and unlikely to have significant clinical implication on peak bone mass for adolescents with an adequate BW. Loss of BW or reduction of BMI was not associated with net loss of aBMD, but individuals who lost weight during follow‐up, demonstrated a slowed progression of aBMD accretion compared with those gaining weight, especially among boys. Considering that more than one of five adolescents was classified as overweight or obese at baseline and with an increasing prevalence during follow‐up for girls and boys, the bone health perspective must be compared with other health benefits. However, adequate weight is important for bone and our results indicate that underweight adolescent boys may benefit from a BMI increase. Particularly underweight individuals losing weight during this critical period of bone accretion could be at risk of a less than optimal peak bone mass acquisition, thus not achieving their full genetic potential for skeletal mass. Because of the short follow‐up of 2 years, results must be interpreted with caution. Further analyses should also examine the effect of lifestyle factors present at baseline. Moreover, the cohort should be followed into adulthood to further explore factors that can alter the bone mass trajectory.

## Disclosures

All authors state that they have no conflicts of interest.

## Supporting information

Supporting Information.

Supporting Information.

## References

[1] Kanis JA , Oden A , McCloskey EV , et al. A systematic review of hip fracture incidence and probability of fracture worldwide. Osteoporos Int. 2012;23(9):2239–56. 22419370 10.1007/s00198-012-1964-3 PMC3421108

[2] Sogaard AJ , Holvik K , Meyer HE , et al. Continued decline in hip fracture incidence in Norway: a NOREPOS study. Osteoporos Int. 2016;27(7):2217–22. 26902091 10.1007/s00198-016-3516-8

[3] Cooper C , Westlake S , Harvey N , Javaid K , Dennison E , Hanson M . Review: developmental origins of osteoporotic fracture. Osteoporos Int. 2006;17(3):337–47. 16331359 10.1007/s00198-005-2039-5

[4] Weaver C , Gordon C , Janz K , et al. The National Osteoporosis Foundation's position statement on peak bone mass development and lifestyle factors: a systematic review and implementation recommendations. Osteoporos Int. 2016;27(4):1281–386. 26856587 10.1007/s00198-015-3440-3 PMC4791473

[5] Rizzoli R , Bianchi ML , Garabedian M , McKay HA , Moreno LA . Maximizing bone mineral mass gain during growth for the prevention of fractures in the adolescents and the elderly. Bone. 2010;46(2):294–305. 19840876 10.1016/j.bone.2009.10.005

[6] Bailey DA . The Saskatchewan Pediatric Bone Mineral Accrual Study: bone mineral acquisition during the growing years. Int J Sports Med. 1997;18 Suppl 3:S191–4. 9272847 10.1055/s-2007-972713

[7] Heaney RP , Abrams S , Dawson‐Hughes B , et al. Peak bone mass. Osteoporos Int. 2000;11(12):985–1009. 11256898 10.1007/s001980070020

[8] Iwaniec UT , Turner RT . Influence of body weight on bone mass, architecture and turnover. J Endocrinol. 2016;230(3):R115–30. 27352896 10.1530/JOE-16-0089 PMC4980254

[9] Zhao LJ , Liu YJ , Liu PY , Hamilton J , Recker RR , Deng HW . Relationship of obesity with osteoporosis. J Clin Endocrinol Metabol. 2007;92(5):1640–6. 10.1210/jc.2006-0572 PMC1868430 17299077

[10] Dimitri P , Bishop N , Walsh JS , Eastell R . Obesity is a risk factor for fracture in children but is protective against fracture in adults: a paradox. Bone. 2012;50(2):457–66. 21619952 10.1016/j.bone.2011.05.011

[11] Dimitri P , Wales JK , Bishop N . Fat and bone in children: differential effects of obesity on bone size and mass according to fracture history. J Bone Miner Res. 2010;25(3):527–36. 19778184 10.1359/jbmr.090823

[12] Goulding A , Grant AM , Williams SM . Bone and body composition of children and adolescents with repeated forearm fractures. J Bone Miner Res. 2005;20(12):2090–6. 16294262 10.1359/JBMR.050820

[13] Goulding A , Taylor RW , Jones IE , McAuley KA , Manning PJ , Williams SM . Overweight and obese children have low bone mass and area for their weight. Int J Obes Rel Metabol Disord. 2000;24(5):627–32. 10.1038/sj.ijo.0801207 10849586

[14] Goulding A , Jones IE , Taylor RW , Manning PJ , Williams SM . More broken bones: a 4‐year double cohort study of young girls with and without distal forearm fractures. J Bone Miner Res. 2000;15(10):2011–8. 11028455 10.1359/jbmr.2000.15.10.2011

[15] Mosca LN , da Silva VN , Goldberg TB . Does excess weight interfere with bone mass accumulation during adolescence? Nutrients. 2013;5(6):2047–61. 23743968 10.3390/nu5062047 PMC3725492

[16] van Leeuwen J , Koes BW , Paulis WD , van Middelkoop M . Differences in bone mineral density between normal‐weight children and children with overweight and obesity: a systematic review and meta‐analysis. Obes Rev. 2017;18(5):526–46. 28273691 10.1111/obr.12515

[17] Sioen I , Lust E , De Henauw S , Moreno LA , Jimenez‐Pavon D . Associations between body composition and bone health in children and adolescents: a systematic review. Calcif Tissue Int. 2016;99(6):557–77. 27484027 10.1007/s00223-016-0183-x

[18] Nilsen OA , Ahmed LA , Winther A , et al. Changes and tracking of bone mineral density in late adolescence: the Tromso Study, Fit Futures. Arch Osteoporos. 2017;12(1):37. 28389986 10.1007/s11657-017-0328-1 PMC5384951

[19] WHO . Report of the Commission on ending childhood obesity. Geneva, Switzerland: World Health Organization 2016.

[20] Bjornelv S , Lydersen S , Mykletun A , Holmen TL . Changes in BMI‐distribution from 1966–69 to 1995–97 in adolescents. The Young‐HUNT study, Norway. BMC Publ Health. 2007;7(1):279. 10.1186/1471-2458-7-279 PMC2082034 17916233

[21] Shapses SA , Sukumar D . Bone metabolism in obesity and weight loss. Ann Rev Nutr. 2012;32: 287–309. 22809104 10.1146/annurev.nutr.012809.104655 PMC4016236

[22] Jacobsen BK , Eggen AE , Mathiesen EB , Wilsgaard T , Njolstad I . Cohort profile: the Tromso Study. Int J Epidemiol. 2012;41(4):961–7. 21422063 10.1093/ije/dyr049 PMC3429870

[23] GE Healthcare Lunar enCORE‐based x‐ray Bone Densitometer User Manual, Revision 5, LU43616EN. 2010 Retrieved June 13, 2019, from https://customer‐doc.cloud.gehealthcare.com/copyDoc/LU43616v13.4EN/1.

[24] Omsland TK , Emaus N , Gjesdal CG , et al. In vivo and in vitro comparison of densitometers in the NOREPOS study. J Clin Densitom. 2008;11(2):276–82. 18158262 10.1016/j.jocd.2007.10.001

[25] Cole TJ , Lobstein T . Extended international (IOTF) body mass index cut‐offs for thinness, overweight and obesity. Pediatr Obes. 2012;7(4):284–94. 22715120 10.1111/j.2047-6310.2012.00064.x

[26] Petersen AC , Crockett L , Richards M , Boxer A . A self‐report measure of pubertal status: reliability, validity, and initial norms. J Youth Adolesc. 1988;17(2):117–33. 24277579 10.1007/BF01537962

[27] Grimby G , Borjesson M , Jonsdottir IH , Schnohr P , Thelle DS , Saltin B . The “Saltin‐Grimby Physical Activity Level Scale” and its application to health research. Scand J Med Sci Sports. 2015;25 Suppl 4:119–25. 26589125 10.1111/sms.12611

[28] Glymour MM , Weuve J , Berkman LF , Kawachi I , Robins JM . When is baseline adjustment useful in analyses of change? An example with education and cognitive change. Amer J Epidemiol. 2005;162(3):267–78. 15987729 10.1093/aje/kwi187

[29] van Breukelen GJ . ANCOVA versus CHANGE from baseline in nonrandomized studies: the difference. Multivariate Behav Res. 2013;48(6):895–922. 26745598 10.1080/00273171.2013.831743

[30] Graham JW , Olchowski AE , Gilreath TD . How many imputations are really needed? Some practical clarifications of multiple imputation theory. Prevent Sci. 2007;8(3):206–13. 10.1007/s11121-007-0070-9 17549635

[31] Greene DA , Naughton GA . Adaptive skeletal responses to mechanical loading during adolescence. Sports Med. 2006;36(9):723–32. 16937949 10.2165/00007256-200636090-00001

[32] Winther A , Dennison E , Ahmed LA , et al. The Tromso Study: Fit Futures: a study of Norwegian adolescents' lifestyle and bone health. Arch Osteoporos. 2014;9(1):185. 24893722 10.1007/s11657-014-0185-0

[33] Travison TG , Araujo AB , Esche GR , McKinlay JB . The relationship between body composition and bone mineral content: threshold effects in a racially and ethnically diverse group of men. Osteoporos Int. 2008;19(1):29–38. 17660933 10.1007/s00198-007-0431-z PMC2664109

[34] Evensen E , Skeie G , Wilsgaard T , et al. How is adolescent bone mass and density influenced by early life body size and growth? The Tromsø Study: Fit Futures‐a longitudinal cohort study from Norway. JBMR Plus. 2018;2(5):268–80. 30283908 10.1002/jbm4.10049 PMC6139726

[35] Von Thun NL , Sukumar D , Heymsfield SB , Shapses SA . Does bone loss begin after weight loss ends? Results 2 years after weight loss or regain in postmenopausal women. Menopause (New York, NY) 2014;21(5):501–8. 10.1097/GME.0b013e3182a76fd5 PMC5032655 24149920

[36] Shapses SA , Riedt CS . Bone, body weight, and weight reduction: what are the concerns? J Nutri. 2006;136(6):1453–6. 10.1093/jn/136.6.1453 PMC4016235 16702302

[37] Shapses SA , Cifuentes M . Body weight/composition and weight change: effects on bone health In: Holick MF, Nieves JW editors. Nutrition and bone health. 2nd ed. New York: Humana Press 2015 pp. 561–83.

[38] Gagnon C , Schafer AL . Bone Health After Bariatric Surgery. JBMR Plus. 2018;2(3):121–33. 30283897 10.1002/jbm4.10048 PMC6124196

[39] El Ghoch M , Gatti D , Calugi S , Viapiana O , Bazzani PV , Dalle Grave R . The association between weight gain/restoration and bone mineral density in adolescents with anorexia nervosa: a systematic review. Nutrients. 2016;8(12). 10.3390/nu8120769 PMC5188424 27916839

[40] Mengel E , Tillmann V , Remmel L , et al. Extensive BMI gain in puberty is associated with lower increments in bone mineral density in estonian boys with overweight and obesity: a 3‐year longitudinal study. Calcif Tissue Int. 2017;101(2):174–81. 28374175 10.1007/s00223-017-0273-4

[41] Rourke KM , Brehm BJ , Cassell C , Sethuraman G . Effect of weight change on bone mass in female adolescents. J Am Diet Assoc. 2003;103(3):369–72. 12616262 10.1053/jada.2003.50051

[42] Weigle DS . Human obesity. Exploding the myths. Western J Med. 1990;153(4):421–8. PMC1002573 2244378

[43] Rocher E , Chappard C , Jaffre C , Benhamou CL , Courteix D . Bone mineral density in prepubertal obese and control children: relation to body weight, lean mass, and fat mass. J Bone Miner Res. 2008;26(1):73–8. 10.1007/s00774-007-0786-4 18095067

[44] Boot AM , de Ridder MA , Pols HA , Krenning EP , de Muinck Keizer‐Schrama SM . Bone mineral density in children and adolescents: relation to puberty, calcium intake, and physical activity. J Clin Endocrinol Metabol. 1997;82(1):57–62. 10.1210/jcem.82.1.3665 8989233

[45] Frost HM . Obesity, and bone strength and "mass": a tutorial based on insights from a new paradigm. Bone. 1997;21(3):211–4. 9276084 10.1016/s8756-3282(97)00124-5

[46] Bonjour JP , Chevalley T . Pubertal timing, bone acquisition, and risk of fracture throughout life. Endocr Rev. 2014;35(5):820–47. 25153348 10.1210/er.2014-1007

[47] Hyldstrup L , Andersen T , McNair P , Breum L , Transbol I . Bone metabolism in obesity: changes related to severe overweight and dietary weight reduction. Acta Endocrinol (Copenh) 1993;129(5):393–8. 8279220 10.1530/acta.0.1290393

[48] Aksglaede L , Juul A , Olsen LW , Sorensen TIA . Age at Puberty and the emerging obesity epidemic. PloS ONE. 2009;4(12):e8450. 20041184 10.1371/journal.pone.0008450 PMC2793517

[49] Crabtree NJ , Arabi A , Bachrach LK , et al. Dual‐energy X‐ray absorptiometry interpretation and reporting in children and adolescents: the revised 2013 ISCD Pediatric Official Positions. J Clin Densitom. 2014;17(2):225–42. 24690232 10.1016/j.jocd.2014.01.003

[50] Willett JB . Questions and answers in the measurement of change. Rev Res Educ. 2016;15(1):345–422.

[51] Allison PD . Change scores as dependent variables in regression analysis. Sociolog Methodol. 1990:93–114.

[52] Riggs BL , Melton Iii LJ, 3rd , Robb RA , et al. Population‐based study of age and sex differences in bone volumetric density, size, geometry, and structure at different skeletal sites. J Bone Miner Res. 2004;19(12):1945–54. 15537436 10.1359/JBMR.040916

[53] Blake GM , Herd RJ , Patel R , Fogelman I . The effect of weight change on total body dual‐energy X‐ray absorptiometry: results from a clinical trial. Osteoporos Int. 2000;11(10):832–9. 11199186 10.1007/s001980070041

[54] Tothill P , Hannan WJ , Cowen S , Freeman CP . Anomalies in the measurement of changes in total‐body bone mineral by dual‐energy X‐ray absorptiometry during weight change. J Bone Miner Res. 1997;12(11):1908–21. 9383696 10.1359/jbmr.1997.12.11.1908

[55] Hangartner TN , Johnston CC . Influence of fat on bone measurements with dual‐energy absorptiometry. Bone Miner. 1990;9(1):71–81. 2337690 10.1016/0169-6009(90)90101-k

[56] Boot AM , de Ridder MA , van der Sluis IM , van Slobbe I , Krenning EP , Keizer‐Schrama SM . Peak bone mineral density, lean body mass and fractures. Bone. 2010;46(2):336–41. 19833245 10.1016/j.bone.2009.10.003

[57] Walsh JS , Henry YM , Fatayerji D , Eastell R . Lumbar spine peak bone mass and bone turnover in men and women: a longitudinal study. Osteoporos Int. 2009;20(3):355–62. 18629566 10.1007/s00198-008-0672-5

