# Supplementary material for: Body Weight and Body Mass Index Influence Bone Mineral Density in Late Adolescence in a Two‐Year Follow‐Up Study. The Tromsø Study: Fit Futures
Source: JBMR Plus. 2019 Aug 21;3(9):e10195. doi: 10.1002/jbm4.10195 (PMC6808229; doi:10.1002/jbm4.10195)
Supplement: Supplementary file 1 — Supporting Information. [file JBM4-3-na-s001.docx]

**Supplemental table 1** *Characteristics at baseline survey Fit Futures 1 (TFF1) and follow-up survey Fit Futures 2 (TFF2) 2 years later: Continuous variables presented as mean (standard deviation) and categorical variables in percentage.* *The Tromsø Study, Fit Futures.*

|  | Girls | | | | | Boys | | | | |
| --- | --- | --- | --- | --- | --- | --- | --- | --- | --- | --- |
|  | TFF1 | | TFF2 | |  | TFF1 | | TFF2 | |  |
|  | n |  | n |  | *p* | n |  | n |  | *p* |
| Age | 355 | 16.61 (0.387) | 355 | 18.60 (0.40) |  | 296 | 16.60 (0.37) | 296 | 18.65 (0.35) |  |
| Age groups at baseline: 15 | 9 | 2.5 % |  |  |  | 19 | 6.4 % |  |  |  |
| 16 | 294 | 82.8 % |  |  |  | 238 | 80.4 % |  |  |  |
| 17 | 52 | 14.7% |  |  |  | 39 | 13.2 % |  |  |  |
| Height (cm) | 355 | 165.03 (6.48) | 355 | 165.73 (6.57) | <.001 | 296 | 177.25 (6.52) | 296 | 179.08 (6.49) | <.001 |
| Weight (kg) | 355 | 60.37 (10.61) | 355 | 63.08 (11.94) | <.001 | 296 | 69.81 (13.68) | 296 | 75.21 (14.64) | <.001 |
| BMI | 355 | 22.17 (3.76) | 355 | 22.97 (4.18) | <.001 | 296 | 22.18 (3.93) | 296 | 23.42 (4.18) | <.001 |
| Sexual maturation ^a^: Early / Completed | 110 | 31.0 % |  |  |  | 22 | 9.1 % |  |  |  |
| Intermediate / Underway | 165 | 46.5 % |  |  |  | 177 | 72.8 % |  |  |  |
| Late / Barely started | 73 | 20.5 % |  |  |  | 44 | 18.1 % |  |  |  |
| Ethnicity White | 347 | 97.8 % |  |  |  | 291 | 98.3 % |  |  |  |
| Others | 8 | 2.2 % |  |  |  | 5 | 1.7 % |  |  |  |
| Physical activity: Sedentary | 42 | 12.0 % | 47 | 13.3 % |  | 77 | 26.3 % | 81 | 28.4 % |  |
| Moderate | 141 | 39.5 % | 144 | 40.8 % |  | 75 | 25.6 % | 60 | 21.1 % |  |
| Sports | 110 | 30.8 % | 110 | 31.2 % |  | 71 | 24.2 % | 77 | 27.0 % |  |
| Competition | 63 | 17.6 % | 52 | 14.7 % |  | 70 | 23.9 % | 67 | 23.5 % |  |
| Smoking (yes) | 68 | 19.0 % | 102 | 28.5 % | <.001 | 62 | 20.9 % | 114 | 38.5 % | <.001 |
| Snuff use (yes) | 108 | 30.2 % | 152 | 42.5 % | <.001 | 108 | 36.5 % | 142 | 48.0 % | <.001 |
| Alcohol consumption (yes) | 262 | 73.2 % | 336 | 93.9 % | <.001 | 195 | 65.9 % | 272 | 91.9 % | <.001 |
| Diseases known to affect bone ^b^ (yes) | 4 | 1.1 % |  |  |  | 5 | 1.7 % |  |  |  |
| Medication known to affect bone ^c^ (yes) | 8 | 2.2 % |  |  |  | 6 | 2.0 % |  |  |  |
| Hormonal contraceptive use (yes) | 118 | 33.0 % |  |  |  |  |  |  |  |  |
| Oestrogen and progestogens | 105 | 29.3% |  |  |  |  |  |  |  |  |
| Progestogens-only | 13 | 3.6% |  |  |  |  |  |  |  |  |
| aBMD FN (g/cm^2^) | 355 | 1.07 (0.12) | 355 | 1.07 (0.13) | 0.008 | 296 | 1.11 (0.15) | 296 | 1.14 (0.15) | <0.001 |
| aBMD TH, (g/cm^2^) | 355 | 1.06 (0.13) | 355 | 1.07 (0.13) | <0.001 | 296 | 1.12 (0.15) | 296 | 1.14 (0.16) | <0.001 |
| aBMD TB (g/cm^2^) | 355 | 1.14 (0.08) | 355 | 1.16 (0.07) | <0.001 | 296 | 1.18 (0.10) | 296 | 1.23 (0.09) | <0.001 |
| BMC FN (g) | 355 | 4.91 (0.71) | 355 | 4.94 (0.72) | <0.001 | 296 | 5.99 (0.99) | 296 | 6.19 (0.99) | <0.001 |
| BMC TH (g) | 355 | 32.01 (4.84) | 355 | 32.42 (4.95) | <0.001 | 296 | 40.17 (6.64) | 296 | 41.26 (6.86) | <0.001 |
| BMC TB (g) | 355 | 2522.89 (387.38) | 355 | 2600.95 (381.68) | <.001 | 296 | 2963.78 (469.83) | 296 | 3200.96 (476.10) | <0.001 |
| BA FN (cm^2^) | 355 | 4.59 (0.34) | 355 | 4.59 (0.34) | .866 | 296 | 5.38 (0.39) | 296 | 5.41 (0.37) | .003 |
| BA TH (cm^2^) | 355 | 30.15 (2.33) | 355 | 30.20 (2.38) | .068 | 296 | 35.73 (2.47) | 296 | 35.99 (2.51) | <.001 |
| BA TB (cm^2^) | 355 | 2207.37 (233.59) | 355 | 2241.68 (224.95) | <.001 | 296 | 2496.46 (240.06) | 296 | 2598.28 (237.87) | <.001 |
| Time between measurements (years) | 355 | 1.95 (0.20) |  |  |  | 296 | 2.01 (0.23) |  |  |  |

^a^ Sexual maturation in girls: menarche age. Missing n=7 (1.97 %). Categories: Early (<12.5), intermediate (12.5-13.9) and late (> 14). Sexual maturation in boys: Puberty Developmental Scale. Categories: Have not begun (<2), barely started (2-2.9), underway (3-3.9) and completed (4). ^b^ Diseases known to affect bone (ICD10): E03 Hypothyroidism, E10 Diabetes type 1. F50.9 Eating disorders, K90.0 Celiac disease and M13 Arthritis. ^c^ Medication known to affect bone (ATC): D07A Plain corticosteroids, H03A Thyroid preparations, N03A Antiepileptic, R01AD Corticosteroids, R03BA Glucocorticoids (inhalants), and H02A Corticosteroids for systemic use.
