# Supplementary material for: Body Weight and Body Mass Index Influence Bone Mineral Density in Late Adolescence in a Two‐Year Follow‐Up Study. The Tromsø Study: Fit Futures
Source: JBMR Plus. 2019 Aug 21;3(9):e10195. doi: 10.1002/jbm4.10195 (PMC6808229; doi:10.1002/jbm4.10195)
Supplement: Supplementary file 2 — Supporting Information. [file JBM4-3-na-s002.docx]

**Supplemental table 2** *Sensitivity analysis of crude models. Comparing regression coefficients for baseline- and changes in weight parameters during follow-up with and without baseline adjustments in femoral neck (FN) and total hip (TH) models. The Tromsø Study, Fit Futures.*

| Girls | | | | | | | | | | | | | | | | | | |
| --- | --- | --- | --- | --- | --- | --- | --- | --- | --- | --- | --- | --- | --- | --- | --- | --- | --- | --- |
|  |  | *∆*aBMD | | | | | | | | | | *∆*BMC | | | | | | |
|  | Baseline adjustment | FN  (n=355) | | | TH  (n=353) | | | TB  (n=355) | | | | FN  (n=355) | | TH  (n=354) | | | TB  (n=355) | |
|  |  | | β | p | | β | *p* | | β | *p* | β | | *p* | β | *p* | β | | *p* |
| Baseline body weight | unadjusted | | 0.003 | 0.134 | | 0.002 | 0.191 | | **-0.001** | **0.246** | **0.009** | | **0.355** | **0.100** | **0.089** | **-16.315** | | **0.010** |
|  | adjusted | | 0.003 | 0.099 | | 0.003 | 0.116 | | **0.002** | **0.184** | **0.024** | | **0.029** | **0.171** | **0.013** | **5.562** | | **0.559** |
| ∆ Body weight | unadjusted | | 0.004 | 0.062 | | 0.005 | 0.006 | | 0.002 | 0.058 | **0.018** | | **0.067** | 0.213 | <0.001 | 52.203 | | <0.001 |
|  | adjusted | | 0.004 | 0.057 | | 0.005 | 0.005 | | 0.002 | 0.026 | **0.026** | | **0.008** | 0.221 | <0.001 | 54.730 | | <0.001 |
| Baseline BMI | unadjusted | | 0.001 | 0.602 | | 0.002 | 0.230 | | **-0.002** | **0.048** | 0.001 | | 0.879 | 0.081 | 0.173 | **-18.844** | | **0.003** |
|  | adjusted | | 0.001 | 0.546 | | 0.002 | 0.335 | | **0.000** | **0.925** | 0.009 | | 0.378 | 0.112 | 0.076 | **-7.260** | | **0.335** |
| ∆ BMI | unadjusted | | 0.001 | 0.538 | | 0.004 | 0.035 | | 0.001 | 0.200 | 0.013 | | 0.177 | 0.175 | 0.003 | 52.960 | | <0.001 |
|  | adjusted | | 0.001 | 0.560 | | 0.004 | 0.030 | | 0.002 | 0.110 | 0.015 | | 0.125 | 0.181 | 0.002 | 54.627 | | <0.001 |

| Boys | | | | | | | | | | | | | | | | | | | |
| --- | --- | --- | --- | --- | --- | --- | --- | --- | --- | --- | --- | --- | --- | --- | --- | --- | --- | --- | --- |
| *∆*aBMD | | | | | | | | | | | | *∆*BMC | | | | | | | |
|  | Baseline adjustment | FN  (n=296) | | | TH  (n=296) | | | | TB  (n=296) | | | FN  (n=296) | | | TH  (n=296) | | | TB  (n=296) | |
|  |  | | β | p | | β | *p* | β | | *p* | β | | *p* | β | | *p* | β | | *p* |
| Baseline body weight | unadjusted | | 0.006 | 0.016 | | 0.006 | 0.016 | **0.001** | | **0.688** | **0.018** | | **0.368** | 0.232 | | 0.049 | **3.407** | | **0.706** |
|  | adjusted | | 0.008 | 0.005 | | 0.008 | 0.005 | **0.006** | | **0.002** | **0.059** | | **0.009** | 0.072 | | 0.023 | **33.515** | | **0.008** |
| ∆ Body weight | unadjusted | | 0.008 | 0.010 | | 0.008 | 0.002 | 0.009 | | <0.001 | 0.067 | | 0.001 | 0.552 | | <0.001 | 94.428 | | <0.001 |
|  | adjusted | | 0.007 | 0.015 | | 0.008 | 0.002 | 0.008 | | <0.001 | 0.064 | | 0.001 | 0.548 | | <0.001 | 93.669 | | <0.001 |
| Baseline BMI | unadjusted | | 0.003 | 0.309 | | 0.005 | 0.049 | **0.000** | | **0.990** | **0.023** | | **0.264** | 0.174 | | 0.140 | **0.583** | | **0.949** |
|  | adjusted | | 0.006 | 0.076 | | 0.006 | 0.021 | **0.005** | | **0.021** | **0.659** | | **0.002** | 0.222 | | 0.082 | **16.358** | | **0.130** |
| ∆ BMI | unadjusted | | 0.003 | 0.290 | | 0.005 | 0.079 | 0.006 | | 0.001 | 0.033 | | 0.106 | 0.350 | | 0.004 | 78.477 | | <0.001 |
|  | adjusted | | 0.003 | 0.333 | | 0.004 | 0.083 | 0.006 | | 0.001 | 0.031 | | 0.120 | 0.347 | | 0.005 | 77.863 | | <0.001 |

*aBMD =Areal bone mineral density (g/cm2), BMC = Bone mineral content (g), FN = Femoral neck, TH = Total hip, BMI= Body mass index (kg/cm^2^), body weight in kg, ∆= change. All* β *coefficients are per SD change in exposure. All coefficients are per SD change in exposure. Disagreement between models in bold*
